# Simple and High Yielding Method for Preparing Tissue Specific Extracellular Matrix Coatings for Cell Culture

**DOI:** 10.1371/journal.pone.0013039

**Published:** 2010-09-27

**Authors:** Jessica A. DeQuach, Valeria Mezzano, Amar Miglani, Stephan Lange, Gordon M. Keller, Farah Sheikh, Karen L. Christman

**Affiliations:** 1 Department of Bioengineering, University of California San Diego, La Jolla, California, United States of America; 2 Department of Medicine, University of California San Diego, La Jolla, California, United States of America; 3 McEwen Centre for Regenerative Medicine, University Health Network, Toronto, Ontario, Canada; The University of Akron, United States of America

## Abstract

**Background:**

The native extracellular matrix (ECM) consists of a highly complex, tissue-specific network of proteins and polysaccharides, which help regulate many cellular functions. Despite the complex nature of the ECM, *in vitro* cell-based studies traditionally assess cell behavior on single ECM component substrates, which do not adequately mimic the *in vivo* extracellular milieu.

**Methodology/Principal Findings:**

We present a simple approach for developing naturally derived ECM coatings for cell culture that provide important tissue-specific cues unlike traditional cell culture coatings, thereby enabling the maturation of committed C2C12 skeletal myoblast progenitors and human embryonic stem cells differentiated into cardiomyocytes. Here we show that natural muscle-specific coatings can (i) be derived from decellularized, solubilized adult porcine muscle, (ii) contain a complex mixture of ECM components including polysaccharides, (iii) adsorb onto tissue culture plastic and (iv) promote cell maturation of committed muscle progenitor and stem cells.

**Conclusions:**

This versatile method can create tissue-specific ECM coatings, which offer a promising platform for cell culture to more closely mimic the mature *in vivo* ECM microenvironment.

## Introduction

The extracellular matrix (ECM) is well known to regulate cell growth [1], [2], survival and maturation/differentiation [3], [4], as well as play an important role in development [5]. Despite the complex nature of the ECM, *in vitro* studies traditionally assess cell behavior on coatings primarily consisting of single purified protein components or directly on polystyrene tissue culture dishes. These surfaces do not mimic the complexity of the extracellular microenvironment and place further limitations on translating findings from *in vitro* studies to the *in vivo* setting [1], [6]. Furthermore, the advent of stem cell research has underscored the importance of extracellular cues for the efficient differentiation and maturation of progenitor cells. Therefore, it is important to provide a growth platform that will allow cells to not only maintain as much of their native morphology and function as *in vivo*, but also the ability to fully mature, particularly in the case of progenitor and stem cells. Deriving and utilizing the native ECM from specific adult tissues could provide an ideal growth platform since it could more appropriately emulate the mature, tissue specific *in vivo* ECM microenvironment.

Typically, single purified matrix proteins from various animal sources are adsorbed to cell culture substrates to provide a protein coating for cell attachment, which may lead to modifications of cellular behavior as cells lose their normal ECM microenvironment. More complex coatings have been used, such as combinations of single proteins of various collagens, fibronectin, vitronectin or laminin, and while these combinatorial signals have been shown to affect cell behavior [7], [8], they do not completely recapitulate the *in vivo* ECM in terms of the tissue-specific combinations and/or ratios of various proteins and polysaccharides. Cell-derived matrices have also been used, such as fibroblast treated plates [9], [10], [11], [12], [13] and Matrigel™ [14] coatings, but they do not mimic any specific native tissue composition. Also, fibroblast pretreated culture platforms have the disadvantage that fibroblasts need to be cultured for several days to deposit matrix [13], and then removed using trypsin prior to using the substrate for cell culture [11], [13]. In general, cells tend to maintain better function on cell-derived matrices, thus providing support for a more *in vivo*-like approach.

While many ECM components are similar, each tissue or organ has a unique composition [15], [16], [17], and a tissue-specific source could better mimic the extracellular microenvironment, thus allowing for tissue-specific cellular development and maturation. The use of tissue-specific coatings has been recently explored [18], [19]; however, limited to no effect was seen on cellular differentiation and morphology when comparing these tissue specific coatings to a conventional collagen coating. In this case, the processing to obtain the material coatings resulted in removal of glycosaminoglycans (GAGs) [18] and potentially other important ECM components. GAGs are sugar residues linked to the core protein of proteoglycans [20], and have been shown to be pivotal for myoblast differentiation [21], [22]. Therefore their omission could have resulted in the little differences observed.

We have previously reported the use of tissue derived ECM as an injectable scaffold for myocardial tissue engineering [23]. In this work, we present a simple method to generate abundant tissue-specific substrate coatings, which retain a complex mixture of ECM proteins, peptides, and GAGs. We further demonstrate the biological activity of the ECM coatings to sustain and promote cell differentiation using two cell culture models: differentiation of the C2C12 mouse myoblast cell line and maturation of human stem cell derived cardiomyocytes. Our results show that these coatings promote committed muscle progenitor differentiation and stem cell maturation *in vitro* when compared to cells grown on traditional cell culture coatings.

## Methods

All experiments in this study were performed in accordance with the guidelines established by the Animal Care and Use Program at the University of California, San.

Diego and the American Association for Accreditation of Laboratory Animal Care, and were approved by the Institutional Animal Care and Use Committee at UCSD (A3033-01).

### Decellularization of skeletal and cardiac tissue for matrix coatings

Hearts and skeletal muscle from the intercostal muscles were harvested from approximately 30–45 kg pigs, which were anesthetized with ketamine (25 mg/kg) and xylazine (2 mg/kg) followed by euthanasia with Pentobarbital (90 mg/kg). Connective tissue and fat was removed from the skeletal muscle, and the major vessels and atria were removed from the heart. The tissue was then cut into ∼1 cm^3^ pieces, and decellularized as previously published [23]. Briefly, the tissue was rinsed with deionized water and then stirred in 1% (wt/vol) solution of sodium dodecyl sulfate (SDS) in phosphate buffered saline (PBS) for 4–5 days. Decellularized skeletal muscle and cardiac tissue was stirred overnight in deionized water to remove the detergent. A sample of decellularized matrix was frozen in Tissue Tek O.C.T. freezing medium, sectioned into 10 µm slices, and stained with hematoxylin and eosin (H&E) to confirm the absence of cells. Following the decellularization protocol, the ECM was lyophilized overnight and milled using a Wiley Mini Mill to create a fine-grained powder.

### Preparation of solubilized skeletal muscle and cardiac matrix for coating

In order to obtain a suspension appropriate for coating plates, the milled form of the matrix was solubilized through enzymatic digestion [23], [24]. Pepsin (SIGMA, St. Louis, MO) was dissolved in 0.1 M hydrochloric acid (HCl) to make a concentration of 1 mg/ml. Approximately 10 mg of the ECM was digested in 1 mL of pepsin solution under constant stirring. After approximately 48 hours, the matrix was diluted using 0.1 M acetic acid to make a 1–2.5 mg/ml concentration of skeletal muscle and cardiac ECM solutions. These solutions were used to coat tissue culture polystyrene for 1 h at 37°C, followed by rinsing with PBS.

### Characterization of skeletal muscle and cardiac matrix

Retained biochemical cues were confirmed using assays for protein and peptide content (SDS-PAGE, mass spectrometry), and polysaccharide content (Blyscan). Solubilized skeletal muscle and cardiac matrix solutions were analyzed by SDS-PAGE and compared to rat tail collagen type I (BD Biosciences, San Jose, CA), as previously described [23]. To determine sulfated glycosaminoglycan (GAG) content, the Blyscan assay (Biocolor, United Kingdom) was used per manufacturer's instructions. Samples were run in triplicate and averaged. Rat tail collagen served as a negative control for the glycosaminoglycan content determination. To more fully characterize the protein content of the cardiac matrix and skeletal matrix, tandem mass spectroscopy (MS/MS) was performed. Matrix samples were digested using trypsin and analyzed by liquid chromatography (LC)-MS/MS with electrospray ionization. A QSTAR-Elite hybrid mass spectrometer (AB/MDS Sciex) that is interfaced to a nanoscale reversed-phase high-pressure liquid chromatograph (Tempo) using a 10 cm-180 ID glass capillary packed with 5-µm C18 ZorbaxTM beads (Agilent). The buffer compositions were as follows. Buffer A was composed of 98% H2O, 2% acetonitrile (can), 0.2% formic acid, and 0.005% trifluoroacetic acid (TFA); buffer B was composed of 100% ACN, 0.2% formic acid, and 0.005% TFA. Peptides were eluted from the C-18 column into the mass spectrometer using a linear gradient of 5–60% Buffer B over 60 min at 400 ul/min. LC-MS/MS data were acquired in a data-dependent fashion by selecting the 4 most intense peaks with charge state of 2 to 4 that exceeds 20 counts, with exclusion of former target ions set to “360 seconds” and the mass tolerance for exclusion set to 100 ppm. Time-of-flight MS were acquired at *m/z* 400 to 1600 Da for 1 s with 12 time bins to sum. MS/MS data were acquired from *m/z* 50 to 2,000 Da by using “enhance all” and 24 time bins to sum, dynamic background subtract, automatic collision energy, and automatic MS/MS accumulation with the fragment intensity multiplier set to 6 and maximum accumulation set to 2 s before returning to the survey scan. Peptide identifications were made using paragon algorithm executed in Protein Pilot 2.0 (Life Technologies). Proteins were labeled based on at least one identified peptide with the confidence of above 99% for that peptide identification.

### Confirmation of cardiac and skeletal muscle matrix adsorption

1 mg/ml cardiac matrix, skeletal muscle matrix, or collagen suspended in 0.1 M acetic acid was adsorbed onto tissue culture plastic for 1 h at 37°C, followed by rinsing with PBS. The total protein and peptide adsorption content was measured using a micro BCA assay (Pierce, Rockford, IL). The amount of adsorbed protein and peptide was determined by calculating the difference in the coating solutions pre- and post-coating.

The presence of GAGs on the coated tissue culture plastic was confirmed by staining the surfaces with toluidine blue (0.15 mg/mL in 50 mM sodium acetate) for 10 minutes [25]. After incubation with toluidine blue, the cardiac matrix, skeletal muscle matrix, collagen, and uncoated plates were rinsed with PBS and assessed for blue staining with brightfield microscopy.

Time of flight secondary ion mass spectroscopy (ToF-SIMS) was also utilized to confirm adsorption of the complex matrices. Tissue culture dishes were coated with skeletal muscle matrix, cardiac matrix, or collagen at 1 mg/ml for 1 h at 37°C, rinsed twice with dH_2_0, dried, and then sent to Tascon USA, Inc. (Chestnut Ridge, NY) for analysis. ToF-SIMS spectra were acquired on an ION-TOF ToF.SIMS 5-300 spectrometer with a 25 keV liquid metal ion source using Bi_3_^+^ primary ions in the bunched mode to achieve high mass resolution (Tascon USA, Inc., Chestnut Ridge, NY). Spectra were acquired for positive secondary ions over a mass range of m/z from 0–800 for evaluation of peak intensities of amino acids fragment ions. The target current used was 0.15 pA, and the area analyzed was approximately 150×150 um^2^. Mass resolution for a typical spectrum was approximately 6000 at m/z = 44 u (C_2_H_6_N^+^). Two reads were performed on two samples for each of the coatings, and two reads were measured on an uncoated tissue culture plate for reference. To emphasize differences in the coatings, relative peak intensities were normalized to the intensity of C2H3+.

### Cell Culture

C2C12 skeletal myoblasts (ATCC, Manassas, VA) were maintained on collagen coated plates in growth media consisting of DMEM (Gibco, Grand Island, NY) supplemented with 10% Fetal Bovine Serum (FBS) and 1% penicillin/streptomycin (Gibco, Grand Island, NY), and split 1∶8 using trypsin when 80% confluent. Myoblasts were diluted to a concentration of 10^7^ cells/ml, and 100 ìl was placed in wells in a 96 well plate pre-coated with either skeletal muscle matrix or collagen, in triplicate, to achieve 100% confluency. At 24 h, non-attached cells were removed with a change of growth media. Cells were cultured in growth media for the extent of the experiment in order to analyze differences in differentiation on the surface coating without any induction media.

Human embryonic stem cells (hESCs, HES2, ESI International) were differentiated into beating embryoid bodies as previously described [26] and maintained after Day 18 of differentiation in induction medium (IM) consisting of Stempro34 (Gibco, Grand Island, NY), 1% glutamine, 150 ug/ml transferrin (Roche, Indianapolis, IN), 50 ng/ml L-Ascorbic Acid (SIGMA), 42 ng/ml monothioglycerol (SIGMA), 10 ng/ml hVEGF (R&D systems, Minneapolis, MN), 5 mg/ml bFGF (R&D systems, Minneapolis, MN). At 35 and 112 days post differentiation, beating embryoid bodies were dissociated by incubation with collagenase Type I (SIGMA, St. Louis, MO) containing 10 ug/ml DNase (Calbiochem, San Diego, CA) for 1 h, followed by washing in Iscove's Modified Dulbecco's Medium (IMDM) and incubation with trypsin/EDTA (SIGMA, St. Louis, MO) for 10 min. Trypsin was inactivated with FBS containing 30 mg/ml of DNase. After incubation with these enzymes mechanical dissociation of embryoid bodies was achieved by drawing the cell suspension through a 20-gauge syringe needle 4–5 times. Cells were then washed in IMDM and pelleted at 1500 rpm for 5 min. Cell pellets were resuspended in IM media and plated onto cardiac matrix or gelatin coated plates, in triplicate, at a density of 1−3×10^5^ cells per 35 mm dish (0.1% solution of porcine gelatin, SIGMA, St. Louis, MO)

### Immunohistochemistry

C2C12 skeletal myoblasts were fixed with 4% paraformaldehyde, incubated with anti-myosin (skeletal, fast) heavy chain (MHC) (1∶200, SIGMA, St, Louis, MO), and then stained with a secondary antibody (1∶200; Invitrogen, Carlsbad, CA) and Hoechst 33342 (1 ìg/ml Invitrogen, Carlsbad, CA). hESC cultures were fixed with 4% paraformaldehyde and incubated with primary antibodies against titin M8 (1∶100, kind gift from Dr. M. Gautel, King's College London, UK), sarcomeric a-actinin (1∶300, A7811, SIGMA, St. Louis, MO), and desmoplakin (1∶100, 2722–5204 AbD Serotec, UK). Cells were then stained with secondary antibodies (1∶250; Jackson Immunoresearch, PA) and Hoechst 33342 stain (1 µg/ml, Invitrogen, Carlsbad, CA), followed by visualization of signals by confocal imaging. Image analysis was performed with Axio-Vision image processing software. Percent differentiation of myoblasts was determined as the number of nuclei in MHC positive cells divided by the total number of nuclei. Five evenly spaced measurements were taken along each myotube to determine myotube width. Average number of nuclei per MHC positive myotube was also determined. For hESC derived cardiomyocytes, average cardiomyocyte cluster area was measured by determining the area of titin M8 positive clusters, and nuclei per cluster was recorded. Average desmoplakin size was determined by measuring pixel area of individual desmoplakin clusters as an indicator of desmosome formation.

### Statistical analysis

All data is presented as the mean ± standard error. For the Blyscan assay, samples were run in triplicate and results averaged, and reported using standard deviation. Significance was determined using a two-tailed student's t-test, and reported as *p<0.05 and **p<0.01.

## Results

### Fabrication of matrix coatings

**Figure 1:**
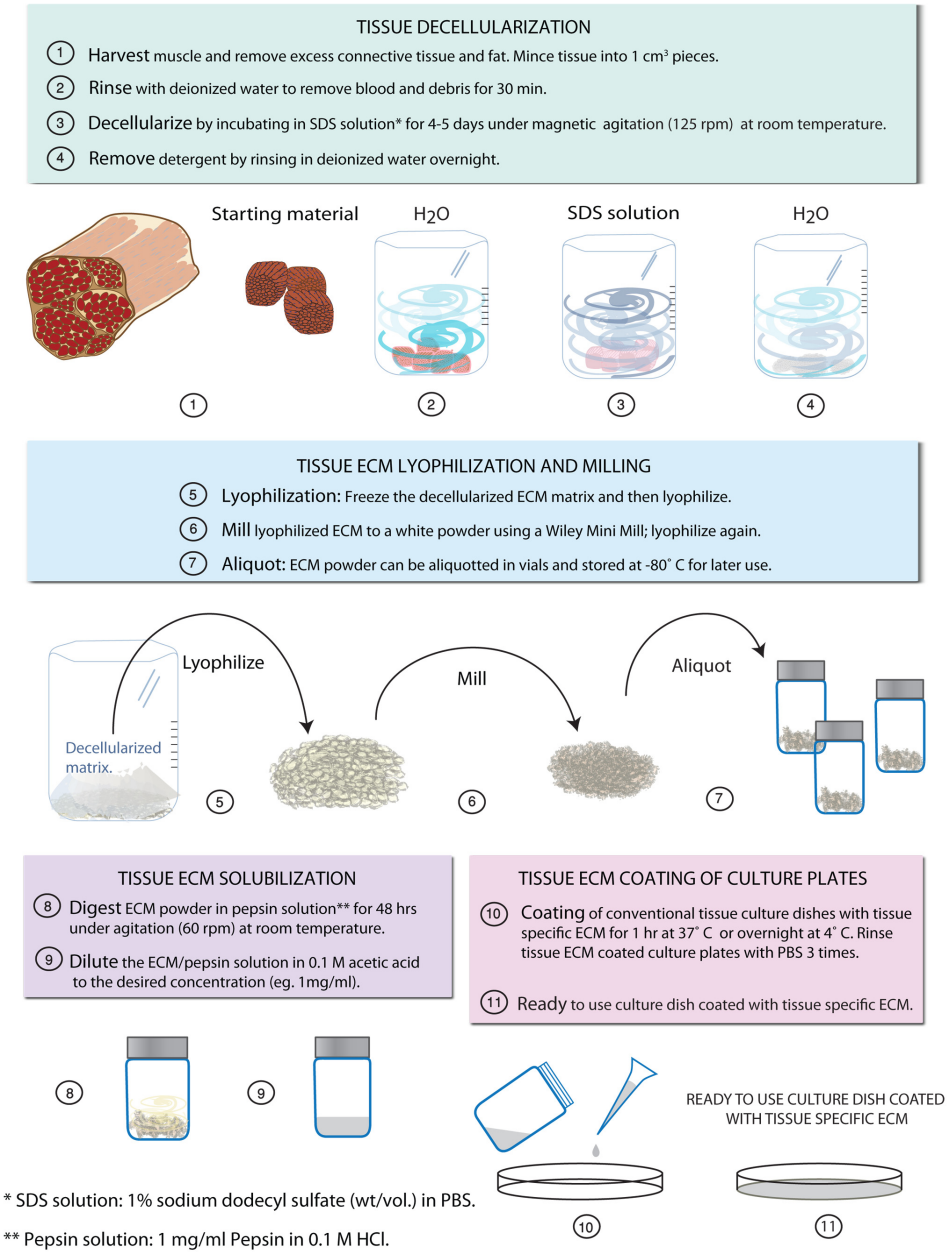
Schematic for the generation of tissue-specific muscle ECM coatings for *in vitro* culture.

**Figure 2:**
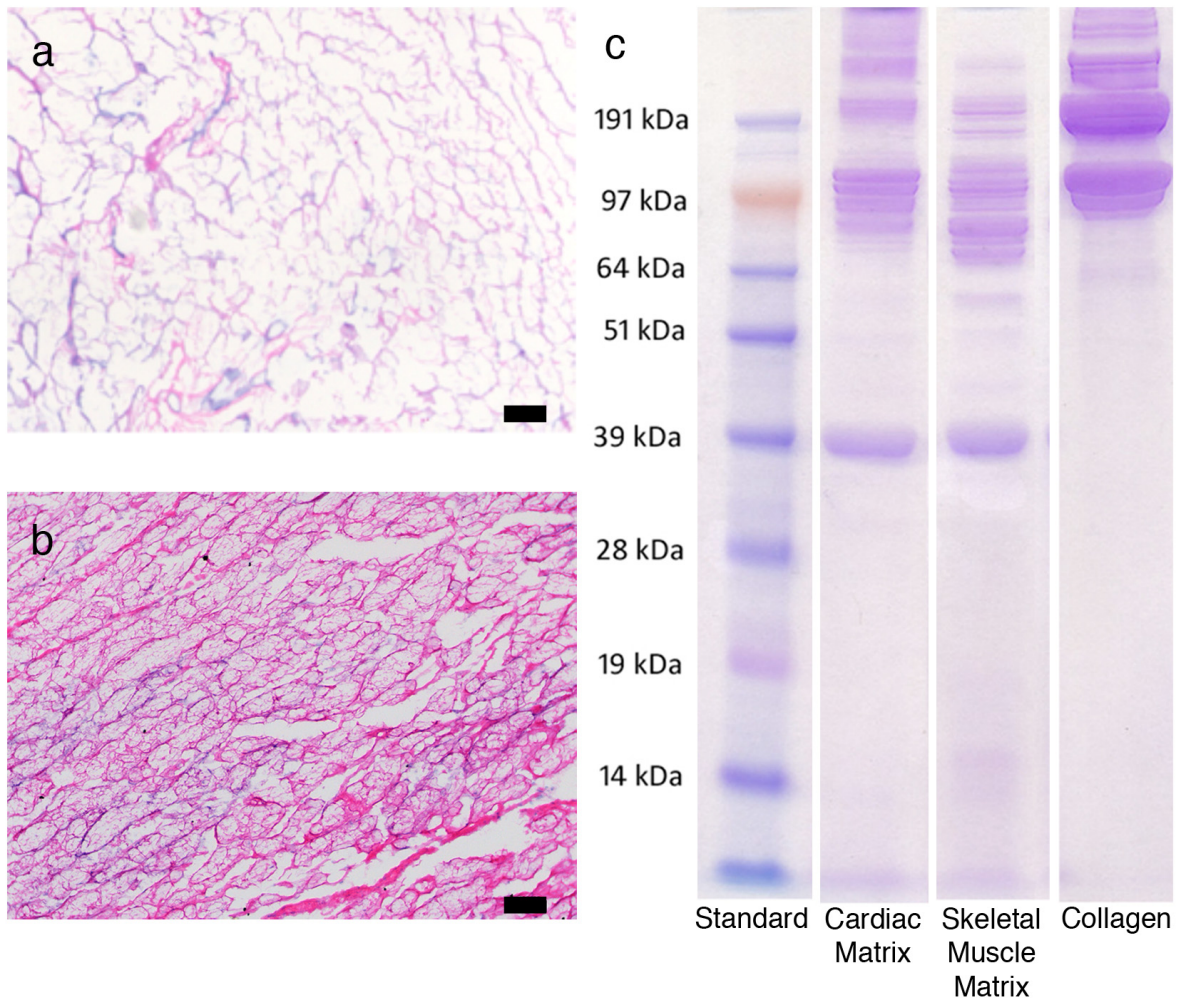
Characterization of cardiac and skeletal muscle matrix. (**a**) H&E section of decellularized cardiac ECM (**b**) H&E section of decellularized skeletal muscle ECM (**c**) PAGE separation of the digested samples compared to collagen I. Note the more complex protein and peptide composition in the muscle matrices.

In order to fabricate the decellularized matrix coatings, ECM was extracted from adult tissues and then processed to create a solubilized mixture of native ECM components that was adsorbed onto tissue culture plastic for cell culture. Figure 1 shows the simplified protocol to achieve solubilized ECM from striated muscle tissue. We have used the same protocol to obtain solubilized ECM from a variety of other tissues including liver and brain (data not shown). To isolate the matrix from adult porcine organs, tissues were decellularized using the detergent sodium dodecyl sulfate (SDS). Adult skeletal and cardiac muscle of porcine origin was cut into cubes approximately 1 cm^3^, rinsed briefly using deionized water, and decellularized with 1% SDS in phosphate buffered saline (PBS) until tissue was completely white and then rinsed thoroughly to remove detergents. Tissue sections were frozen, sectioned and then stained using hematoxylin and eosin (H&E), as shown by an absence of nuclei (Figure 2a,b). After confirmation of decellularization, the matrix was lyophilized and milled into a fine powder, solubilized through enzymatic digestion using pepsin [23], [24], and kept at acidic pH to prevent self-assembly (Figure 1).

### Characterization of solubilized decellularized matrix

Assays for biochemical composition demonstrated that the solubilized muscle matrix coatings retained complex biochemical cues, including proteins, peptides, and polysaccharides. As a first step in demonstrating that the solubilized matrix was composed of a complex milieu of proteins and peptides, we performed PAGE. The solubilized matrices exhibited bands that corresponded to collagen, as expected, in addition to the presence of lower molecular weight bands (Figure 2c). We also performed more detailed characterization through mass spectroscopy to identify the components within the material. Interestingly, the results indicated that the components of cardiac matrix originated from a mixture of collagens ranging from collagen I to collagen VI, as well as elastin, fibrinogen, fibronectin and laminin, components expected to be found in the myocardial ECM. In addition, fibrillin-1, a glycoprotein found in elastic fibers, and lumican, a keratan sulfate proteoglycan, as well as fibulin-3 and -5 were identified (Table 1). Skeletal muscle matrix components contained a mixture of collagen I, II, V, VI, VIII, XI, XII, and XIII. Mass spectroscopy also identified fibrinogen, fibrillin-1, fibulin-5, and the proteoglycans lumican, dermatopontin, and decorin. Proteoglycans can influence cell behavior, and dermatopontin and decorin in particular play a role in matrix assembly, growth factor signaling and myoblast differentiation [27], [28], [29], [30]. Furthermore, our solubilized ECM contained heparan sulfate (HS) GAGs, which play an important role processes such as development and angiogenesis [31], [32], and are also implicated in muscle regeneration [21]. However, this methodology is not all-inclusive and, therefore, there may be other extracellular matrix components that were not identified. Using a Blyscan assay we also found that our process allowed for the retention of GAGs, which have been shown to be vital for skeletal myoblast differentiation [21], [22]. The solubilized skeletal muscle matrix contained 16.7±0.1 µg GAG per mg of matrix, while the cardiac matrix had approximately 20 µg GAG per mg of matrix as previously reported [23]. The retention of tissue specific components demonstrates that while the 3D structure of the original ECM is lost with this method, many of the original biochemical cues are retained and thus these materials may mimic the subtle complexities of each tissue's ECM.

**Table 1:** Mass spectroscopy composition analysis.

	Cardiac Matrix	Skeletal Muscle Matrix
Collagen I	X	X
Collagen II	X	X
Collagen III	X	
Collagen IV	X	
Collagen V	X	X
Collagen VI	X	X
Collagen VIII		X
Collagen XI		X
Collagen XII		X
Collagen XIII		X
Elastin	X	
Fibrinogen	X	X
Fibronectin	X	
Laminin	X	
Fibrillin-1	X	X
Lumican	X	X
Fibulin-3	X	
Fibulin-5	X	X
Heparan Sulfate		X
Dermatopontin		X
Decorin		X

### Surface characterization of ECM coatings

To ensure that the muscle matrices could be adsorbed onto tissue culture plastic, we quantified the amount of protein/peptide adsorbed, verified the presence of GAGs on the surface, and assessed compositional differences using ToF-SIMS. The amount of protein that adsorbed to the tissue culture plastic was measured using a micro BCA assay, which calculates the remaining protein/peptide content in the coating solutions. The difference in total protein/peptide in solution pre- vs. post-adsorption was calculated to be 17.4±1.5 µg/ml for skeletal muscle matrix and 27.4±1.3 µg/ml for cardiac matrix, with collagen measuring at 18.35±0.5 µg/ml. This translates roughly to 0.05-0.09 µg protein adsorbed per mm^2^ of tissue culture plastic. Additionally, GAG adsorption to the tissue culture plastic was demonstrated by incubating the coated surfaces with toluidine blue [33]. The cardiac and skeletal matrix coated surfaces remained blue after PBS rinse, whereas the collagen and uncoated dishes were clear (data not shown).

**Figure 3:**
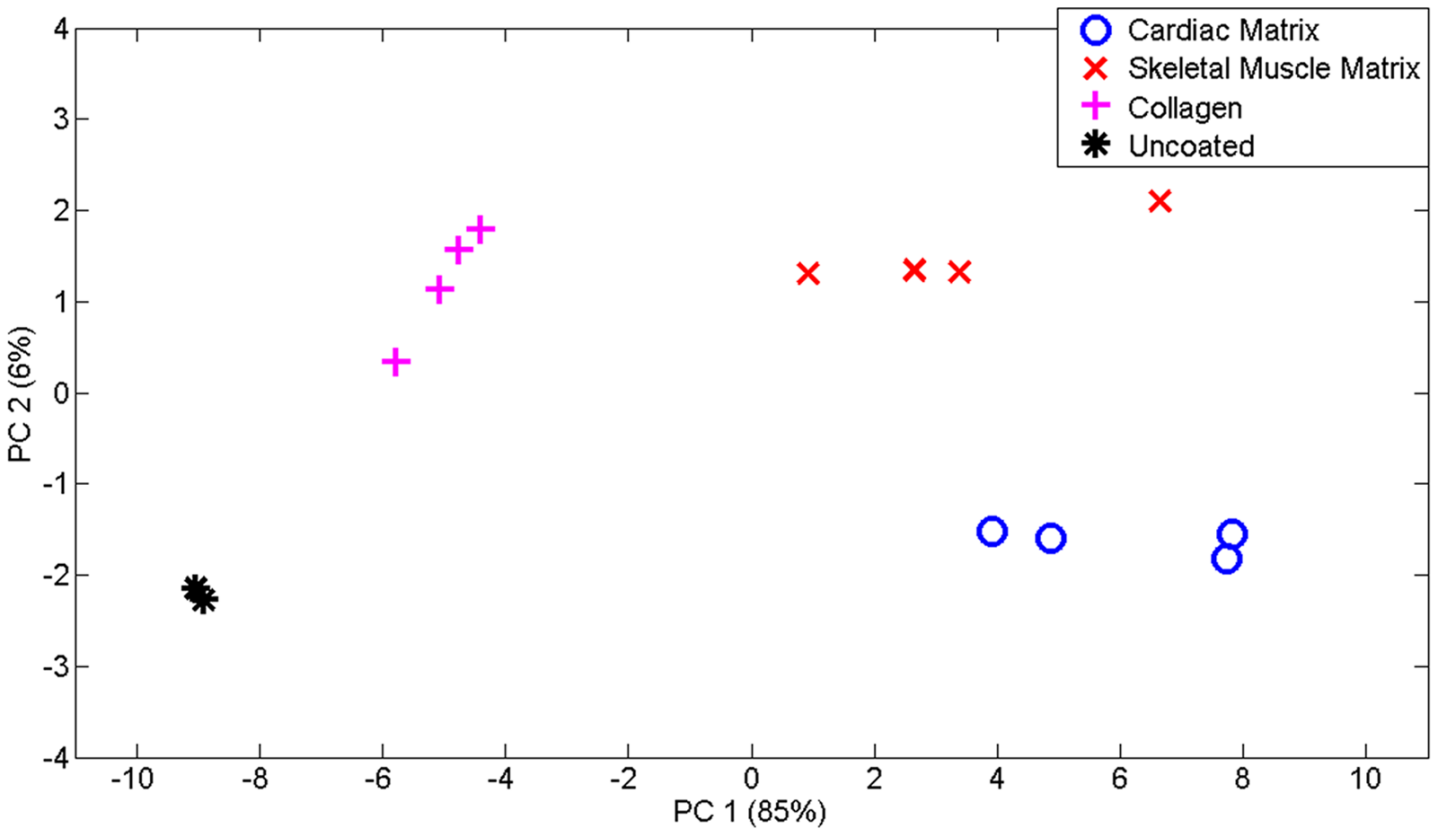
Principal component analysis of surface composition differences as detected using ToF-SIMS. PC1 versus PC2 scores plots for cardiac matrix-, skeletal matrix-coated tissue culture plastic versus collagen coated and uncoated controls. Distinct differences are observed between the coatings as well as the controls, indicating that both of the solubilized muscle matrices adsorbed to the surface.

We further employed ToF-SIMS (time of flight secondary ion mass spectroscopy) to analyze cardiac matrix- and skeletal matrix-coated polystyrene dishes compared to collagen-coated and uncoated controls. A previously published peak list [34], which includes peak characteristics of unique amino acid mass fragmentation patterns created from a model of ECM protein surfaces, was chosen for ToF-SIMS. These peaks correspond to amino acids, which are matched to common ECM proteins [34], [35], [36] (Table 2). Principal Component Analysis (PCA) was used to highlight any differences in the matrix coatings; PCA is a mathematical analysis technique that can be used to characterize the differences between a large set of data by calculating the principal components (PC) [37]. The analysis revealed that there existed distinct differences between the compositions of cardiac matrix-, skeletal muscle matrix-, collagen-coated surfaces, and the uncoated control due to the clustering of the data when PC2 was graphed against PC1 (Figure 3). Most of these differences occurred along PC1 (85%) while PC2 accounted for 6% of the differences.

**Table 2:** ToF-SIMS composition analysis (adapted from [34], [35], [36]).

Peak Number	Molecular Fragment	Prevalent Amino Acid	Extracellular Matrix Association
1	CH_4_N	Gly	Collagen I
2	C_2_H_4_NO	Gly	Collagen I
3	C_2_H_5_S	Met	Collagen I
4	C_4_H_6_N	Pro	Collagen I
5	C_3_H_6_NO	Gly	Collagen I
6	C_3_H_5_N_2_O	Gly	Collagen I
7	C_4_H_10_SN	Met	Collagen I
8	C_4_H_5_N_2_O_2_	Gly	Collagen I
9	C_4_H_7_N_2_O_2_	Gly	Collagen I
10	C_5_H_9_SO	Met	Collagen I
			
11	C3H3O	Tyr	Fibronectin
12	C2H6NO	Ser	Fibronectin
13	C4H5O	Thr	Fibronectin
14	C3H3O2	Ser	Fibronectin
15	C4H10N	Val	Fibronectin
16	C3H8NO	Thr	Fibronectin
17	C5H7O	Val	Fibronectin
18	C4H6NO	Glu	Fibronectin
19	C7H7O	Tyr	Fibronectin
20	C8H10NO	Tyr	Fibronectin
21	C10H11N2	Trp	Fibronectin
22	C11H8NO	Trp	Fibronectin
			
23	CH3N2	Arg	Laminin
24	CH5N3	Arg	Laminin
25	C3H4NO	Asn	Laminin
26	C2H7N3	Arg	Laminin
27	C2H6SN	Cys	Laminin
28	C4H4NO2	Asn	Laminin
29	C4H10N3	Arg	Laminin
30	C4H11N3	Arg	Laminin
31	C5H11N4	Arg	Laminin
			
32	C2H6N	Ala, Cys	
33	C4H5N2	His	
34	C5H10N	Lys	
35	C3H7N2O	Asn, Gly	
36	C3H6NO2	Asp, Asn	
37	C5H7N2	His	
38	C5H8N3	His, Arg	
39	C8H10N	Phe	
40	C6H5N2O	His	
41	C9H8O	Phe	

**Figure 4:**
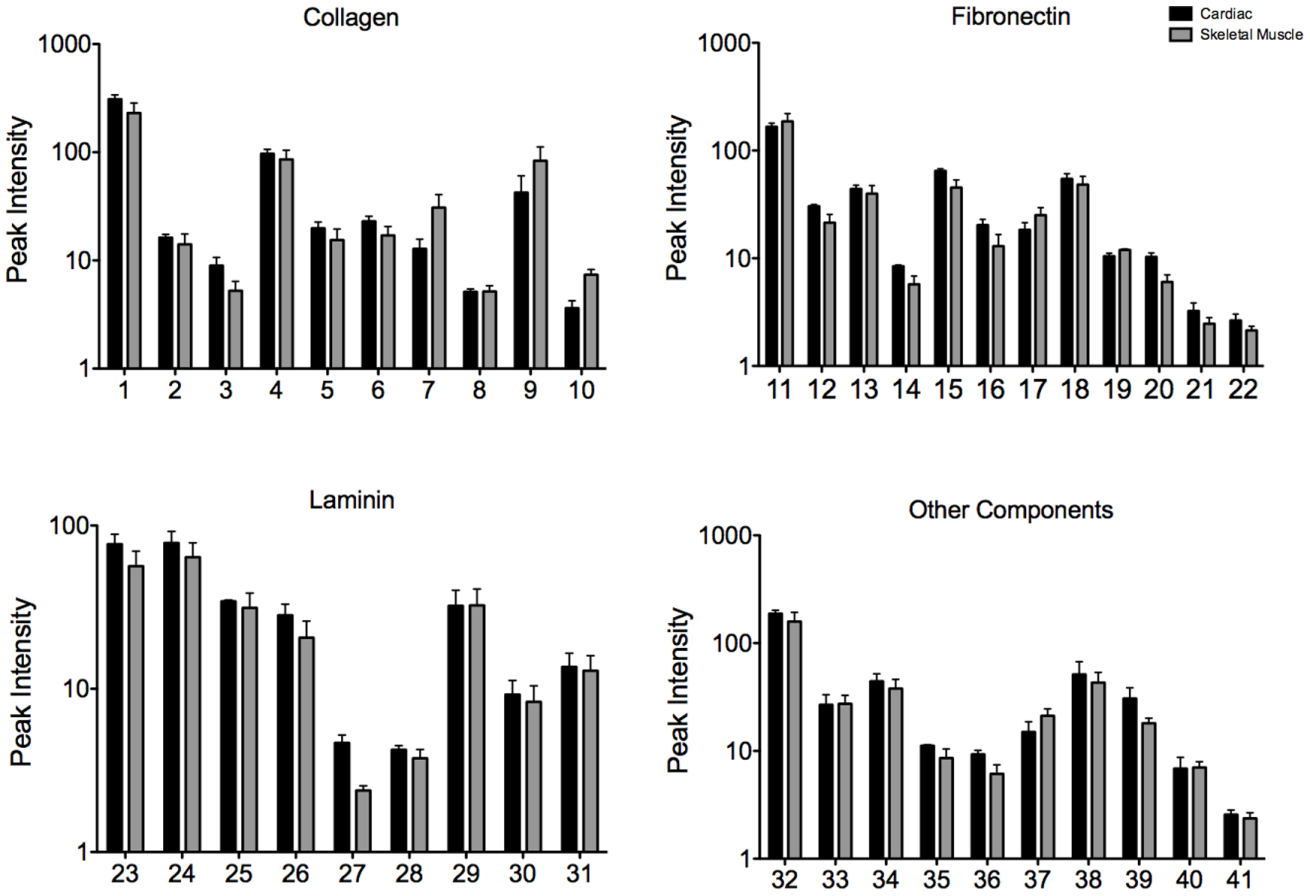
Peak value data of functional groups of cardiac matrix and skeletal muscle matrix coatings. ToF-SIMS peak analysis of cardiac matrix versus skeletal muscle matrix coated tissue culture plastic. The amino acid signatures from the peak values indicate that post adsorption the different matrices retain their complexity with multiple ECM components. See Table 2 for individual peak descriptions.

To further demonstrate that the muscle matrix coatings were more complex than simply collagen and GAGs, we examined individual peaks based on the corresponding amino acid and ECM protein. Both matrix coatings contained peaks associated with amino acids found in fibronectin and laminin as well as collagen (Figure 4). Thus, these studies confirmed that the tissue specific coatings adsorbed to the surface of tissue culture plastic, and were more complex than collagen. Moreover, cardiac matrix and skeletal muscle matrix coatings were distinct from each other.

### Enhanced C2C12 cell differentiation on skeletal matrix coating

**Figure 5:**
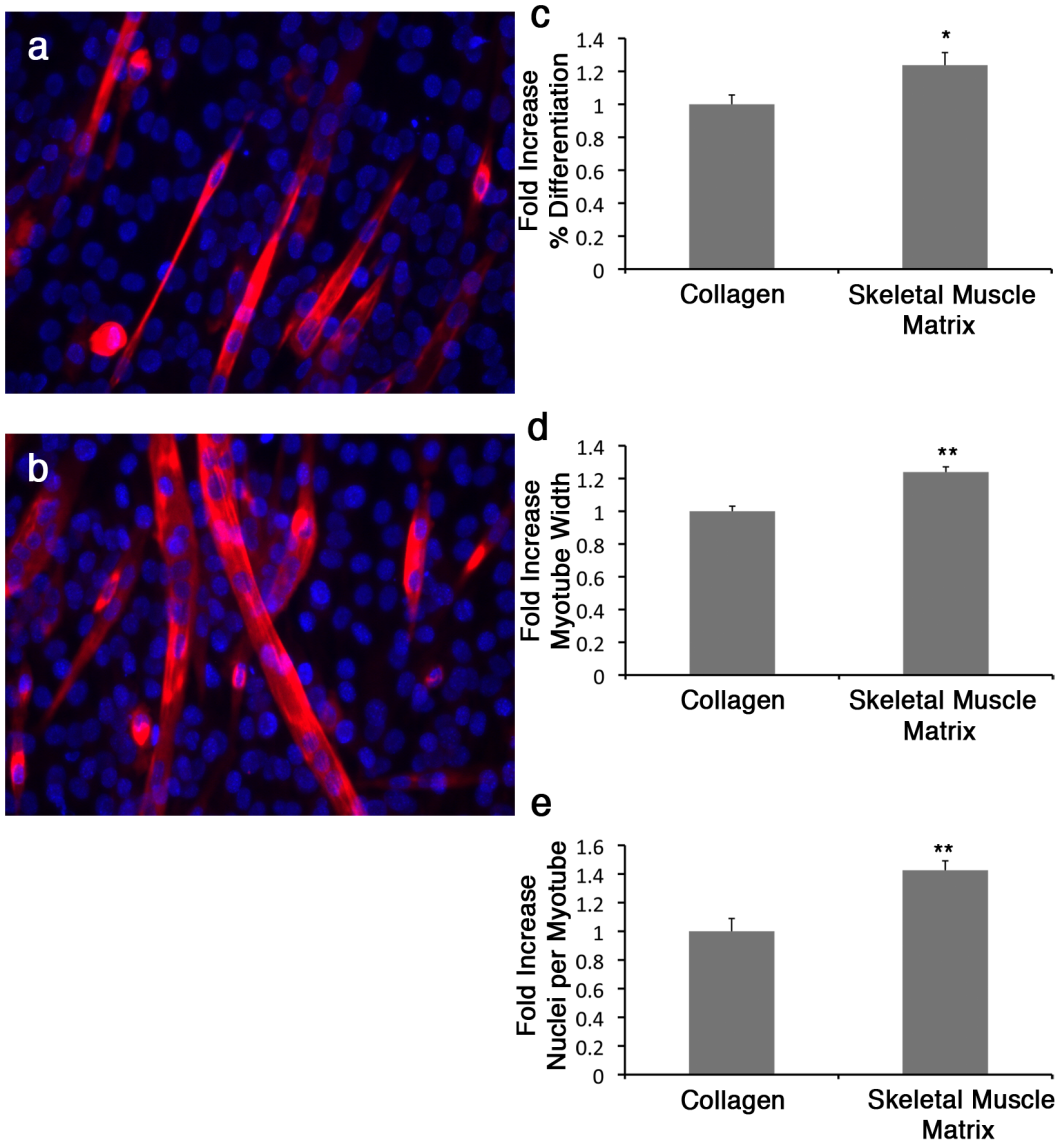
C2C12 differentiation/maturation is increased on skeletal muscle matrix coating when compared to standard collagen I. C2C12 myotube formation is shown at Day 3 on collagen I (**a**) or skeletal muscle matrix (**b**), as labeled by myosin heavy chain (red) staining, and DNA labeled with Hoechst 33342 (blue). Percent differentiation (**c**), myotube width (**d**), and nuclei per myotube (**e**) were significantly increased when myoblasts were grown on the skeletal muscle matrix coating compared to the conventional collagen coating. *p<0.05, **p<0.01.

C2C12 skeletal myoblasts are a well-characterized myogenic cell line capable of differentiation by fusion into multinucleated myotubes [38]. These cells were expanded in growth media, without any components to induce differentiation, and then plated on culture plates coated with either skeletal muscle matrix or collagen. The number of myotubes at 7 days of differentiation was unchanged in cells grown on solubilized native ECM as compared to collagen (data not shown). However C2C12 myoblasts cultured on native ECM differentiated earlier (day 3, p<0.05), and had increased fiber diameter and fusion index (p<0.01) (Figure 5). Figure 5 displays images of myotube formation from day 3 after plating. As the main component in both coatings was collagen, the formation of larger, more nucleated myotubes and increased earlier differentiation on the tissue specific matrix illustrates that ECM components other than collagen in the skeletal muscle matrix retained biological activity and have an important effect on C2C12 skeletal myoblast differentiation.

### Enhanced hESC-derived cardiomyocytes cell maturation on cardiac matrix coating

**Figure 6:**
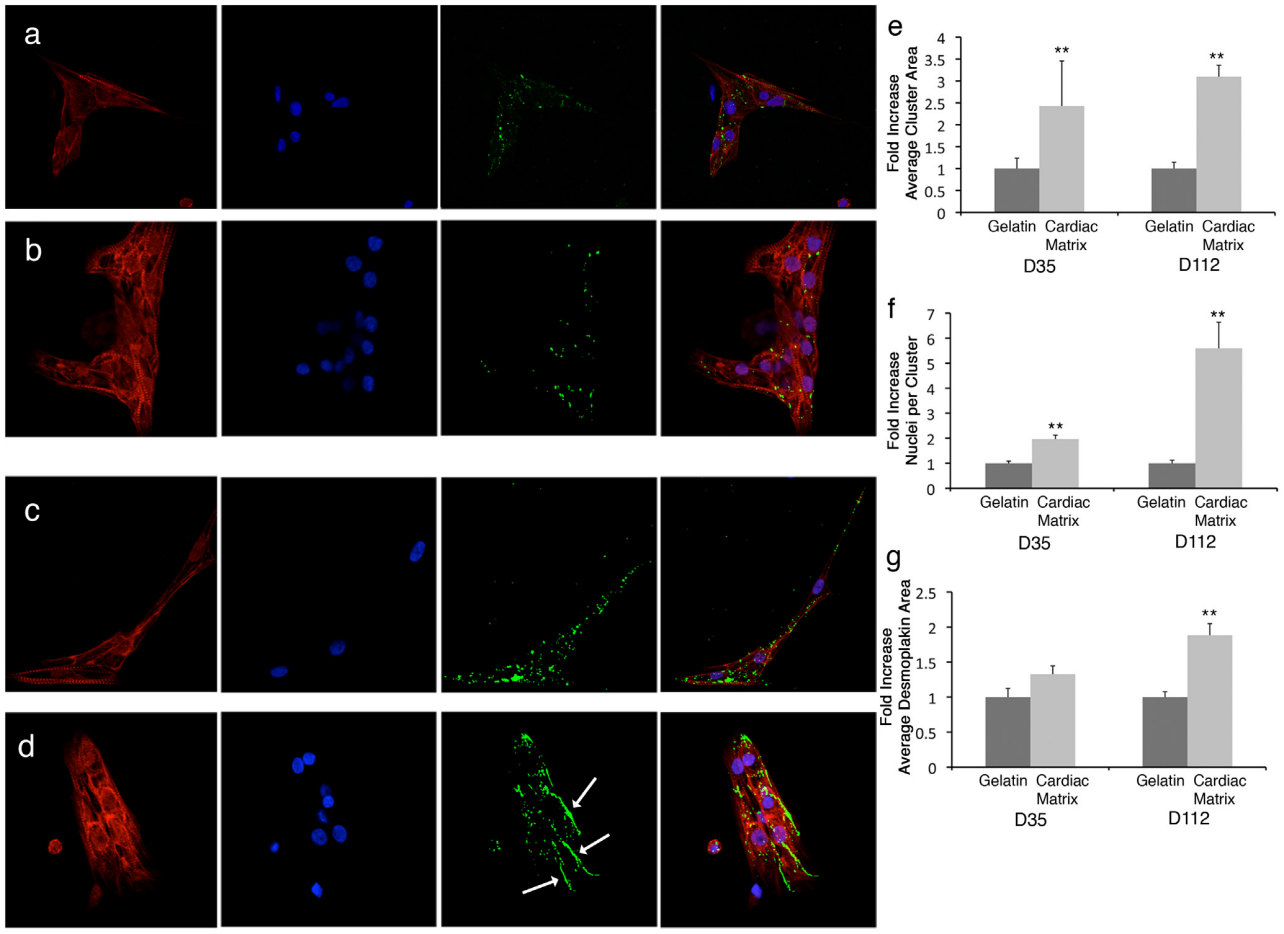
Cardiac matrix coatings increase hESC derived cardiomyocyte maturation compared to the standard gelatin substrate. hESC-derived cardiomyocytes were cultured on gelatin (**a** and **c**) or cardiac matrix (**b** and **d**) and stained for Titin M8 (red), desmoplakin (green), or nuclei (blue). Cell cluster area (**e**), number of nuclei per cluster (**f**), and average desmoplakin positive area (**g**) were measured and compared between the two coatings. Cardiomyocytes had an increased cluster area and number of nuclei at both day 35 and 112. At day 112, the average desmoplakin immunoreactive area was significantly greater on the cardiac matrix, with localization observed at the lateral ends of the cardiomyocytes (arrows), indicating the formation of desmosomes and a more mature phenotype. **p<0.01.

While differentiation of hESCs into cardiomyocytes is routinely achieved, maturation beyond a fetal phenotype has not been readily achieved [39], [40], [41], [42]. We therefore pre-committed hESCs to cardiomyocytes and examined whether an adult cardiac matrix coating could further promote maturation of these cells, compared to gelatin. Gelatin was chosen as the comparison coating, as it is the standard substrate for many differentiation protocols with hESCs [26], [43], [44]. Of the mixed population, only cells that were positively stained for Titin M8 were assessed, as Titin M8 is a late cardiac muscle-specific marker. Cells were also stained for desmoplakin, and nuclei. Desmoplakin is a cell-cell junction protein that displays a punctate distribution along the sarcolemma during cardiac muscle development, but eventually localizes to the lateral ends of muscle cells at the intercalated disc to form functional intercellular junction structures known as desmosomes, which are important for cell maturation and mechanical and structural integrity [45], [46], [47]. Desmosomes also participate in morphogenesis and differentiation [48], and we therefore used desmoplakin, a key component of the desmosomal complex, as a marker to assess whether the cardiac matrix coating would promote a more mature phenotype. hESC-CMs displayed striking differences on the cardiac matrix when compared to gelatin (Figure 6), namely an increased multi-cellular organization and maturation. hESC-CMs were found to organize into multi-cellular clusters as indicated by a significant increase in myofibrillar area and number of cardiomyocyte nuclei per cell cluster area when plated on the cardiac matrix compared to gelatin. While there were no differences in the localization of desmoplakin at day 35, at day 112 the immunostaining demonstrated that desmoplakin organized and aggregated to form large localized areas at the lateral/transverse ends between the cardiomyocytes plated on the cardiac matrix, resembling the intercalated disc localization of desmosomal proteins found in fully mature cardiomyocytes *in vivo* [46]. Hence, there was more mature localization of the desmosomal cell-cell junction protein, indicating increased maturation. It should be noted that similar changes in the spatiotemporal patterns of cardiac intercalated disc proteins have been observed during postnatal development and maturation of the human ventricular myocardium [49].

## Discussion

There is a compelling need for the development of coating materials that mimic the native cellular microenvironment for better *in vitro* assays and for the expansion of cells for cell-mediated therapies. In addition, a culture platform where cells are surrounded by an environment that more closely resembles the native ECM niche would improve the assessment of cell behavior in other areas of research such as drug development applications. There has been a shift of cell culture substrates from single purified proteins, to more complex materials that attempt to mimic the complex native ECM [9]. Combinations of purified proteins have been shown to improve cell proliferation and differentiation, demonstrating that complex coatings are beneficial [9], [50]. However, there are many potential combinations, and using a natural matrix would be more physiologically relevant.

In this study, we have presented a method to create matrix coatings derived from porcine adult tissues, performed analysis to determine the components of the solubilized extracellular matrix, and then ensured that the surface composition retained its complexity after adsorption onto tissue culture plastic. We hypothesized that these coatings would have a beneficial effect within 2D muscle culture systems as they more appropriately emulate the native muscle ECM *in vitro*. This culture substrate would thus allow for desirable cell-matrix interactions and provide a better platform for cell phenotype and differentiation, similar to that found *in vivo*. A potential limitation, however, of this approach is that these matrices are of porcine origin, which has the potential for disease transmission and immune recognition. However, the FDA has approved many decellularized xenogeneic ECMs for implantation including porcine dermis, small-intestine submucosa, and heart valves [51] and therefore, we anticipate that these matrices will be a viable option for cell culture. For clinical translation of any cells grown on these coatings, appropriate quality control and testing will of course be essential to ensure lack of pathogens.

The goal of this study was to demonstrate that the simple method for fabricating naturally derived, tissue specific matrix coatings could provide a cell culture substrate that emulates the native ECM microenvironment, is readily available, and is as simple to use as conventional protein coatings. The ECM plays an important role in directing progenitor and stem cell differentiation and maturation [8], [52] and we thus examined the potential for these tissue specific coatings in committed progenitor and stem cell culture. Differentiation of muscle progenitor cells was studied using C2C12 skeletal myoblasts on adult skeletal muscle matrix and compared to a conventional collagen coating I [53]. To examine the potential of the adult myocardial matrix as a coating, we examined its effect on the morphology and maturation of hESC derived cardiomyocytes as compared to the standard gelatin coating typically used in stem cell derived cardiomyocyte cultures [54], [55] since we hypothesized that the adult cardiac matrix coating could further promote maturation of these cells.

Our data shows that the native tissue matrix enhanced differentiation of C2C12 skeletal myoblasts and maturation of human embryonic stem cell derived cardiomyocytes when compared to conventional cell culture coatings. While these extracellular matrix coatings contains many biochemical cues of the native environment, one limitation of this study is that it is likely that some ECM components have been lost during processing. However, the remaining matrix components do affect cell differentiation and maturation. *S*keletal muscle matrix contains native cues that allowed for increased differentiation and larger myotubes to develop from skeletal myoblasts when compared to a standard single protein coating. In addition, the cardiac matrix enhanced the formation and organization of multicellular clusters with intercellular desmosomal like structures resembling the cardiac intercalated disc within hESC derived cardiomyocytes. Thus, this report highlights that the cell microenvironment and subtle differences in matrix composition can play an important role on cell phenotype *in vitro*. We show a method that can allow one to more closely recapitulate this microenvironment in 2D cell culture, which could be applied to any type of cell culture assay. Given that the origin of these matrices is adult ECM, it is likely more applicable to terminal differentiation/maturation and adult cell culture. There is also a potential application for cell culture using other cell types, as to date, we have created coatings from liver, brain, and other muscle tissue and therefore anticipate that this process can be used to generate substrate coatings from any non-mineralized tissue to present the appropriate cell-matrix interactions. 3D culture systems have recently been highlighted as a better mimic of the *in vivo* environment [56], and similar matrix solutions to those employed here as coatings can self assemble into thin films or gels for 3D cell culture [23], [24]. The 3D environment is not, however, always suitable for all culture studies. Herein, we have developed a simple method to produce large amounts of coating material that would provide a better approximation of the *in vivo* microenvironment with the added benefit of tissue specificity and complexity. The results of this study show the potential of decellularized, solubilized extracellular matrix coatings derived from muscle to promote maturation of committed progenitor and stem cells *in vitro*, demonstrating this method's applicability to muscle cell culture; however, the general technology has wide ranging implications.
